# Enhanced Agronomic Traits and Medicinal Constituents of Autotetraploids in *Anoectochilus formosanus* Hayata, a Top-Grade Medicinal Orchid

**DOI:** 10.3390/molecules22111907

**Published:** 2017-11-07

**Authors:** Hsiao-Hang Chung, Shu-Kai Shi, Bin Huang, Jen-Tsung Chen

**Affiliations:** 1 Department of Horticulture, National Ilan University, Yilan City 260, Taiwan; hhchung@niu.edu.tw; 2 Department of Life Sciences, National University of Kaohsiung, Kaohsiung 811, Taiwan; ha998322@gmail.com; 3 Department of Biomedical Science and Environmental Biology, Kaohsiung Medical University, Kaohsiung 807, Taiwan; huangpin2@yahoo.com.tw

**Keywords:** flavonoid, flow cytometry, gastrodin, polyploidy, UPLC-MS/MS

## Abstract

This study developed an efficient and reliable system for inducing polyploidy in *Anoectochilus formosanus* Hayata, a top-grade medicinal orchid. The resulting tetraploid gave a significant enhancement on various agronomic traits, including dry weight, fresh weight, shoot length, root length, leaf width, the size of stoma, and number of chloroplasts per stoma. A reduction of the ratio of length to width was observed in stomata and leaves of the tetraploid, and consequently, an alteration of organ shape was found. The major bioactive compounds, total flavonoid and gastrodin, were determined by the aluminum chloride colorimetric method and ultra performance liquid chromatography tandem mass spectrometry (UPLC-MS/MS), respectively. The tetraploid produced significantly higher contents of total flavonoid and gastrodin in the leaf, the stem, and the whole plant when compared with the diploid. The resulting tetraploids in this study are proposed to be suitable raw materials in the pharmaceutical industry for enhancing productivity and reducing cost.

## 1. Introduction

*Anoectochilus formosanus* Hayata, also known as the Taiwan jewel orchid (2*n* = 2*x* = 24), is a small terrestrial orchid with high medicinal value grown in Taiwan and other countries [1]. This plant is also called “The King of Medicine” or “The Tiger of Medicine” because of its multiple pharmaceutical effects, including protection of liver, anti-inflammatory, treatment of chest and abdominal pains, diabetes, nephritis, fever, cardiovascular diseases, and cancer prevention [2,3,4]. Due to their high economical value, wild Taiwan jewel orchids have been harvested excessively and were threatened. In spite of the importance of medical applications, there are only two reliable tissue culture protocols for mass propagation of this species, mainly via asymbiotic germination and multiple shoot induction from nodal stems, respectively [5,6]. In this present study, we use nodal stems as explants to induce polyplidy via shoot induction. Otherwise, based on our knowledge, limited success was found in studying the physiology, genetics, and plant breeding of this species.

Gastrodin is an effective bioactive compound found in *A. formosanus* and other medicinal orchids [7]. It can promote the secretion of brain-derived neurotrophic factor and thus has considerable effects in clinical treatment of central nervous system disorders, especially the recovery of neurological function and to protect neural cells against injury [7,8]. Flavonoids are plant secondary metabolites, and they have beneficial functions in human health, i.e., the prevention and treatment of different pathologies via a protection from the attack by reactive oxygen species (ROS) or other mechanisms [9]. It has been reported that flavonoid glycosides and their derivatives of *A. formosanus* resulted in strong antioxidant properties that could prevent oxidative stress in human systems and thus have potential usages in cancer chemoprevention [10,11]. Polyploidy induction is a powerful technique to produce enhanced agronomic characteristic for plant breeding and altered genotypes for genetic studies [12,13]. In addition, ploidy manipulations can provide a rapid means or a promising parameter to realize enhanced production of phyto-pharmaceuticals [14,15]. In this present study, an efficient and reliable system for inducing tetraploidy in the Taiwan jewel orchid was established. The tetraploids were selected for determination of gastrodin and total flavonoid to test the effect of the ploidy level on the production of bioactive constituents in this species.

## 2. Results

### 2.1. Induction of Polyploidy

**Figure 1:**
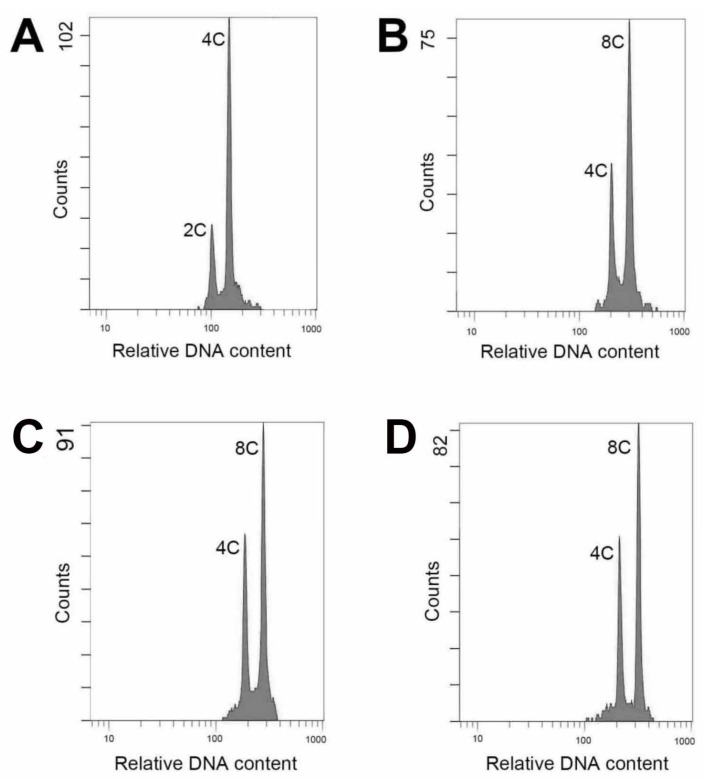
Flow cytometric analysis of *Anoectochilus formosanus*. (**A**) Diploid (wild type); (**B**) Tetraploid (first generation); (**C**) The clonal regenerant of 4*x* plants (tenth generation); (**D**) The seedling of 4*x* plants (first generation).

The nodal-stem explants were used to test the effects colchicine (concentrations at 0, 100, 250, 500 and 1000 mg/L) on polyploidy induction. On a hormone-free medium without the supplement of colchicine, each nodal-stem explant produced approximately 3.0 shoot. However, in the presence of 100–1000 mg/L colchicine, the explant became severely brown and could not produce shoot on the hormone-free medium (data not shown). The supplement of 0.5 mg/L *N*-(Phenylmethyl)-7*H*-purin-6-amine (*N*^6^-benzyladenine, BA) or 0.5 mg/L 1-Phenyl-3-(1,2,3-thiadiazol-5-yl)-urea (thidiazuron, TDZ) enhanced the shoot development from the explant. Despite the application of 0.5 mg/L BA or 0.5 mg/L TDZ, 100–1000 mg/L colchicine significantly retarded the number of surviving shoots (Table 1). In the colchicine treatments, 100 mg/L colchicine plus 0.5 mg/L TDZ gave the highest number of survival shoots (approximately 8.3/explant) and the highest percentage of polyploids (50%) (Table 1). The flow cytometric analysis revealed that the diploid showed a two-peak (2C + 4C) pattern in the relative nuclear DNA content (Figure 1A). By contrast, the tetraploid had a characteristic pattern with 4C and 8C peaks (Figure 1B).

### 2.2. The Stability of Polyploidy

The resulting tetraploids of *A. formosanus* were kept in vitro by subculturing of stem nodal cuttings for more than five years and the clonal regenerants still retained its ploidy level (Figure 1C). The tenth generation of regenerants showed a high purity and stability in their ploidy state and morphological characteristics (Table 2). A high purity of polyploidy was also proved in seedlings from the 4*x* plants, since the cells taken from young leaves, lateral shoots, and root tips all showed a typical 4*x* cytometric pattern (Figure 1D, Table 2). Except for the 2*x* and 4*x* plants, no other ploidy levels or mixploids were found.

### 2.3. Analysis of Agronomic Traits

**Figure 2:**
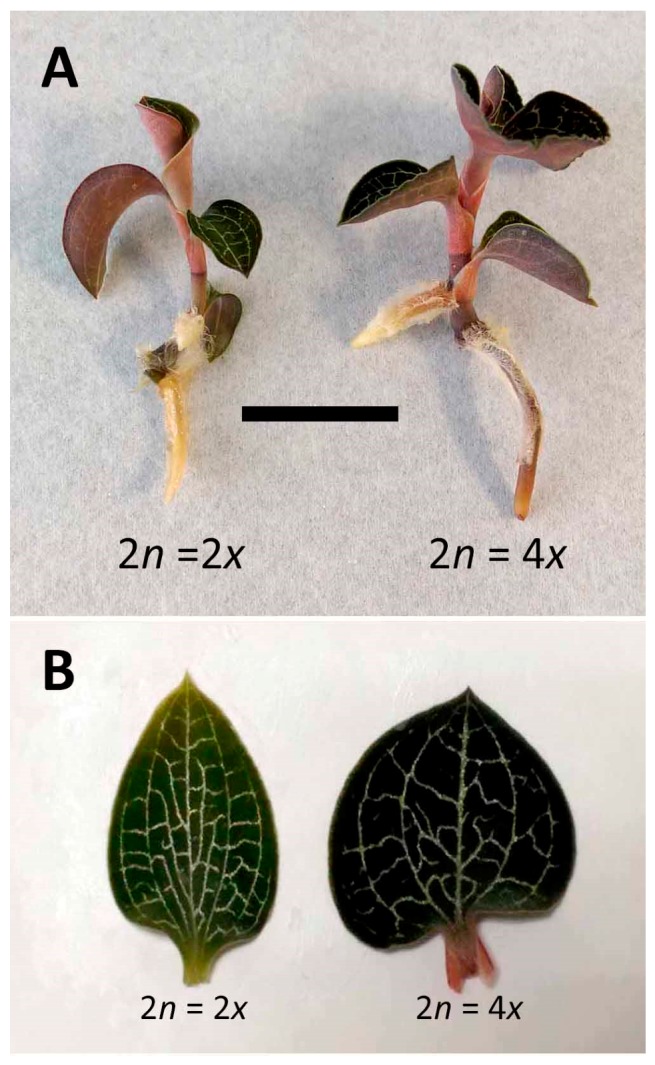
Growth and morphology of 2*x* and 4*x* plantlets in *Anoectochilus formosanus* (The scale bar is fitting for all figures. *Bar* = 2.5 and 1.0 cm for Figure 3A and 3B, respectively). (**A**) Plantlets; (**B**) Leaves.

The tetraploid had significant higher responses in growth performances, including dry weight, fresh weight, shoot length, root length, and leaf width, when compared with the diploid (Table 3). Generally, the size of the tetraploid was obviously larger than the diploid (Figure 2A). The diploid had a significant higher length to width of the leaf than the tetraploid (Table 3). Consequently, the leaf shape was dramatically changed by the ploidy level, and the tetraploid had cordate leaves rather than ovate leaves as the diploid typically (Figure 2B). The leaf blade of the tetraploid was obviously larger than the diploid and a darker color was found in the leaf of the tetraploid (Figure 2B). When transplanted to pots, the tetraploid still maintained a better growth performance than the diploid.

### 2.4. Analysis of Stomata

**Figure 3:**
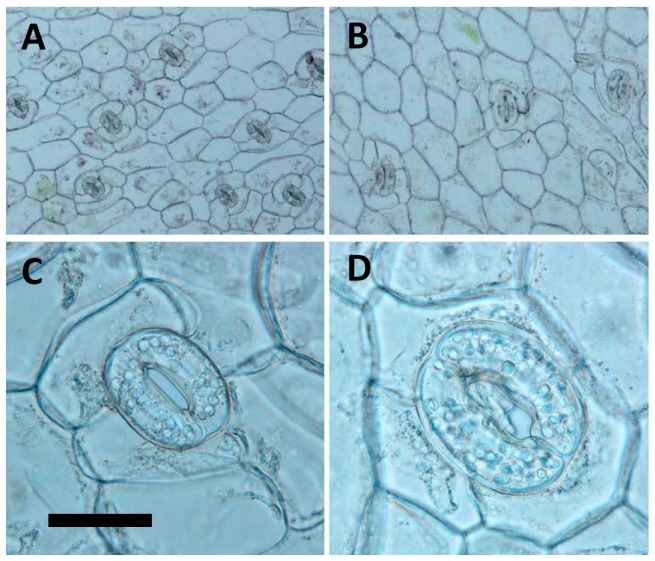
Stomata of 2*x* and 4*x* plants in *Anoectochilus formosanus* (The scale bar is fitting for all figures. Bar = 80 μm for Figure 2A,B, 20 μm for Figure 2C,D, respectively). (**A**) Diploid (magnification = 100×); (**B**) Tetraploid (magnification = 100×); (**C**) Diploid (magnification = 400×); (**D**) Tetraploid (magnification = 400×).

The tetraploid had a significantly lower stomatal frequency and length to width ratio of the stoma when compared with the diploid (Table 4, Figure 3A,B). The tetraploid had significantly higher stoma length and width than the diploid (Table 4). The guard cells and epidermal cells became obviously larger in the tetraploid when compared with the diploid (Figure 3C,D). The tetraploid had a significantly higher number of chloroplasts per stoma (Table 4).

### 2.5. Analysis of the Gastrodin Content

**Figure 4:**
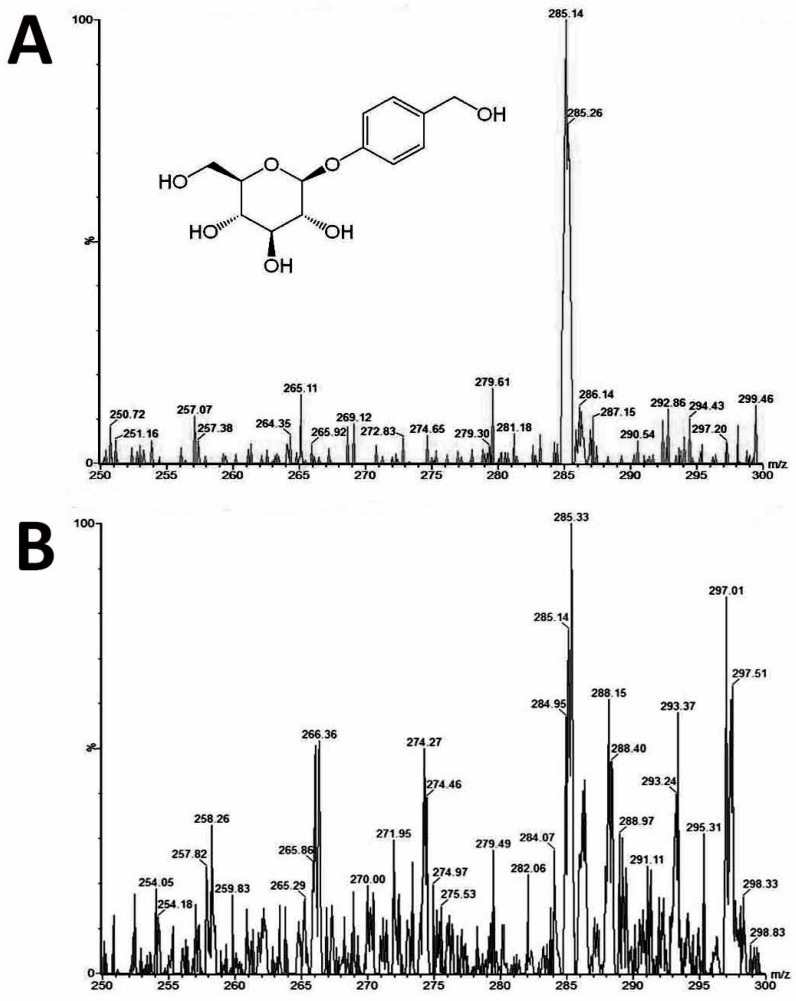
The chemical structure of gastrodin and mass spectra of *Anoectochilus formosanus*. (**A**) The standard gastrodin; (**B**) The extract from a plant sample.

**Figure 5:**
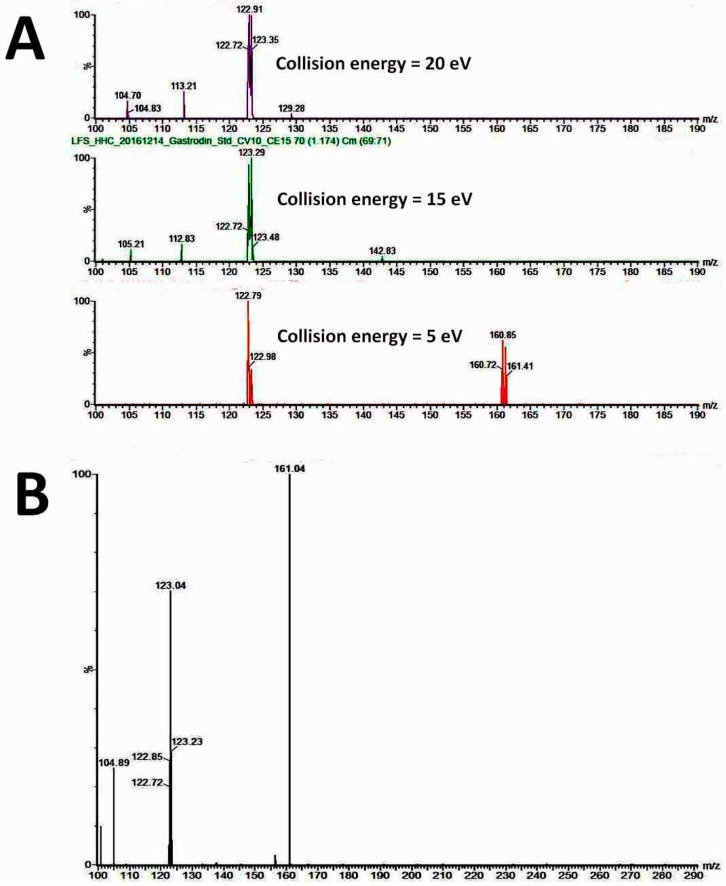
The product ion spectra. (**A**) The standard gastrodin at different collision energy; (**B**) The extract from a plant sample.

**Figure 6:**
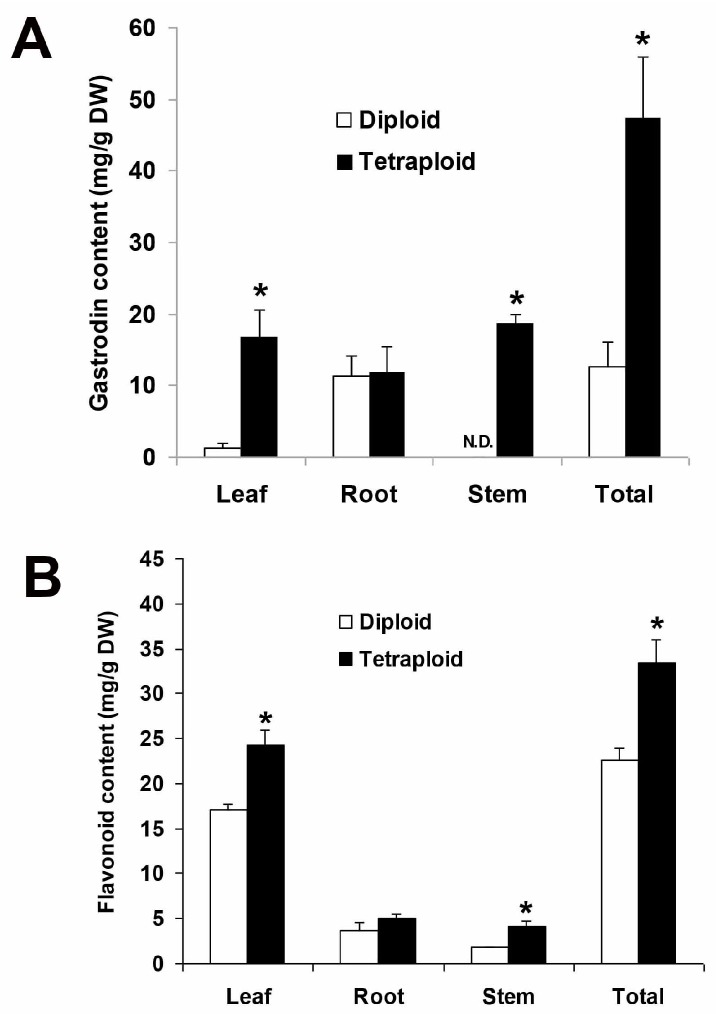
(**A**) The gastrodin content in the diploid and the tetraploid of *Anoectochilus formosanus*; (**B**) The flavonoid content in the diploid and the tetraploid of *Anoectochilus formosanus*. Data are expressed as mean ± SD. * Indicates a significantly difference between the diploid and the tetraploid in leaf, root, stem, and total (the sum of leaf, root, and stem), respectively, according to Duncan’s multiple range test (*P* ≤ 0.05).

The mass-to-charge ratio (*m*/*z*) of the standard gastrodin was 285.14 (Figure 4A). The extract from plant samples of *A. formosanus* also has a peak of *m*/*z* 285.14 that applied as the precursor ion (Figure 4B). To optimize the parameters for analyzing using the product ion, three collision energies (CE) were tested (Figure 5A), and the optimized CE was at 15 eV (Table 5). After the parameter optimization as shown in Table 4, the multiple reaction-monitoring (MRM) transitions *m*/*z* 285.1 → 123 was applied for quantification of gastrodin (Figure 5B). In the diploid, almost all the gastrodin was produced in the root (Figure 6A). However, it is another matter in the tetraploid: the leaf and stem produced higher contents of gastrodin than the root (Figure 6A). The tetraploid gave a significantly higher content in gastrodin of the leaf and the stem when compared with the diploid (Figure 6A). Consequently, the tetraploid had a significantly higher total content of gastrodin in the whole plants (Figure 6A).

### 2.6. Analysis of the Flavonoid Content

Independent of the ploidy level, a highest content of flavonoids was found in the leaf when compared with the root and the stem (Figure 6B). The tetraploid gave a significantly higher content in flavonoids of the leaf and the stem when compared with the diploid (Figure 6B). Consequently, the tetraploid had a significantly higher total content of flavonoids in the whole plants (Figure 6B).

## 3. Discussion

Dealing with polyploidy induction, the explant type together with its resulting regeneration pathway could be crucial factors for the successful induction rate and also the purity of the polyploids [13]. Therefore, for inducing of polyploidy in medicinal plants, various explant types have been used, including apical buds, callus, cotyledonary nodes, leaf explants, seeds, shoots, shoot tips, and zygotic embryos [13,16,17,18]. In this present study, the nodal-stem explants of *A. formosanus* performed well in the polyploidy induction via a three-day treatment of colchicine in the liquid medium. Here, we suggest that this process could increase the exposure area of the shoot bud to the antimitotic agent and thus may reduce chimera.

TDZ was proposed as a multidimensional plant growth regulator that has both auxin and cytokinin effects and could induce a diverse array of in vitro morphogenesis [19,20,21,22,23]. Ket et al. reported that TDZ could induce multiple shoot proliferation of *A. formosanus* [6]. Therefore, we suggest that TDZ was beneficial for polyploidy induction in *A. formosanus* because of its enhancement of the plantlet survival in the presence of colchicine which severely retarded growth of the explants.

The ploidy level of plants could be analyzed using several protocols, including chromosome counting, flow cytometric analysis, morphological observation and evaluation of anatomical parameters [18,24,25,26,27]. It has been reported that flow cytometry is an efficient and reliable protocol and was used to evaluate polyploidy in 63% of previous related publications [13]. In this study, there was a satisfactory performance when evaluated ploidy level using flow cytometry. The technique is efficient and the materials for testing comprise young leaf tissues could be obtained easily and without killing the parent plant.

It was reported that ploidy could affect many morphological and fitness traits, including stomatal size, flower size, seed weight, and biomass [28,29]. In this study, similar marked differences were found in several agronomic traits, including biomass, plant size, leaf shape, and stomatal morphology. In polyploidy selection, the increased leaf size has been considered as one of the remarkable changes in morphology by the ploidy level [30]. The tetraploid of *Lolium* cultivars had a faster leaf elongation rate and consequently longer mature cells than did the diploid [31]. In *A. formosanus*, the tetraploid had a significantly higher leaf width than did the diploid. Consequently, the ratio of leaf length to width in the tetraploid was lower down to approximately 0.95. Therefore, the leaf shape was dramatically changed by the ploidy level, i.e., the leaf shape of the diploid was ovate, but the tetraploid had a cordate leaf. Here we suggest that leaf size and leaf shape could be a reliable indicator for identification of polyploidy.

The morphology of stomata had been documented as a reliable indicator for polyploidy selection [30,32]. In *A. formosanus*, the tetraploid had a dramatically change in frequency, size, the ratio of length to width, and number of chloroplasts of stomata, and therefore the stomatal morphology, could be a reliable selection indicator for polyploidy inducing in the future test.

In theory, the regeneration pathway without the intervention of callus is an efficient method of obtaining regenerants and could reduce unnecessary somaclonal variations [23,33]. In the present study, the 4*x* plants of *A. formosanus* showed a high stability in ploidy level as their tenth generation of clonal regenerants derived from nodal stem cuttings still maintained the polyploidy (2*n* = 4*x*) after five years of subculturing.

Tetraploidy could enhance the production of bioactive compounds via heritable genetic variation or overexpression of relative biosynthesis pathways, e.g., the tropane biosynthetic pathway for scopolamine production in *Hyoscyamus muticus* [34,35]. The tetraploid of *A. formosanus* had a significantly higher content of gastrodin in the leaf, stem, and the whole plants. Therefore, here we suggest that the tetraploid may possess a higher activity of phenolic glycoside biosynthesis and consequently could accumulate a higher content of gastrodin. In previous reports, there were marked differences in flavonoid accumulation in relation to ploidy level [35,36]. A similar result was also found in this present study, the tetraploid had a significantly higher total content of flavonoids than the diploid in *A. formosanus*.

In conclusion, an efficient and reliable system for polyploidy induction of *A. formosanus* was successfully established in this study. The resulting tetraploids showed a significant promotion on various agronomic traits and an alternation on organ shape. In addition, the tetraploid produced significantly higher contents of bioactive compounds, including total flavonoid and gastrodin.

## 4. Materials and Methods

### 4.1. Plant Materials

In vitro-grown clonal *Anoectochilus formosanus* Hayata plants (2*n* = 2*x* = 24) from Winpower Technology Co. (Kaohsiung, Taiwan) were used as donor plants in this study. These plants were maintained in vitro for approximately 3 months, and each plant had approximately 3–4 nodes and 8–10 leaves.

### 4.2. Subculture of the In Vitro–Grown Plants

The in vitro-grown plants were removed the leaves and cut into nodal-stem segments, then propagated on the basal medium (BM) containing full strength medium Murashige and Skoog (1962) medium (MS) [37] salt and vitamins, 30 g/L sucrose and 9 g/L agar. The pH of the media was adjusted to 5.8 with 1M KOH or HCl prior to autoclaving for 20 min at 121 °C. The culture containers were 600-mL flasks, and each contained 100 ml of the medium. The cultures were incubated in a growth chamber with a 16/8 h (light/dark) photoperiod at an irradiance of 42–55 μmol m^−2^ s^−1^ (daylight fluorescent tubes FL-20BR/18, 18 W, (China Electric Co., Taipei, Taiwan)) and a temperature of 25 ± 2 °C. The subculture period is approximately six weeks.

### 4.3. Induction of Polyploidy

The nodal-stem explants (the leaves were removed and each contained three nodes) were used to test the effects of colchicine on polyploidy induction. Various concentrations of colchicine (i.e., 0, 100, 250, 500, and 1000 mg/L) were added to liquid BM for induction of polyploidy. Following three days of induction, the cultures were transferred onto three different media, including hormone-free 0.5 mg/L BA and 0.5 mg/L TDZ-containing solid BM that was devoid of colchicine, to induce shoot development. Following an additional six weeks, all the cultures were transferred to solid BM supplemented with 0.5 mg/L 1*H*-Indole-3-butanoic acid (indole-3-butyric acid, IBA) to obtain plantlets. The light and temperature conditions were the same as mention above. Four replicates (each contains five nodal-stem explants) were performed in each treatment.

### 4.4. Flow Cytometric Analysis

The young leaves of the nodal-stem-derived plantlets, including the wild type and putative polyploids (first generation), were used for identification of the polyploidy. The analysis was performed using the flow cytometer (Cytomics^TM^ FC500, Beckman Coulter Inc., Indianapolis, IN, USA). The fresh leaves (each was 2 × 2 cm^2^) were chopped with a razor blade in a 6 cm glass dish containing 500 μL of the extracting buffer (containing 10 mL/L Triton X-100, 6.3 g/L Na_2_SO_3_ and 50 mL/L 1 M Tris-Hcl) and filtered through a 30 μm nylon mesh. The released nuclei were stained with 10 μL/L of propidium iodide (No. AVK250, Strong Biotech Corp., Taipei, Taiwan) for further analysis. For each sample, approximately 2000 nuclei were analyzed.

### 4.5. Analysis of Stability of Polyploidy

Ploidy level and morphological characteristics (stomata density, length of stomata, width of stomata, and leaf shape) of tenth generation (five years) of clonal plants that derived from in vitro nodal stem cultures and seedlings obtained via self-pollination of 4*x* plants (first generation) were evaluated to prove the purity and stability of their polyploidy. Young leaves, lateral shoots and root tips from 1.5-year-old plants were harvested separately for flow cytometric analysis.

### 4.6. Analysis of Stomata and Agronomic Traits

The fully expanded leaves from the third node of nine-month-old in vitro plantlets (tenth generation) were used to compare the difference in stomata between the 2*x* plants to the 4*x* plants. Data of stomata density and length and width of stomata were collected using Olympus BX41 microscope (Tokyo, Japan) at magnifications of 100×, 400× and 400×, respectively. The epidermis of leaves was peeled for analysis of stomata density at an area of 500 × 500 μm^2^ (at a magnification of 100×). The pictures were taken at a magnification of 100× or 400× with DP20 microscope camera (Olympus, Tokyo, Japan) and application software (DP2-BSW-E, Olympus, Tokyo, Japan). The nine-month-old plantlets (tenth generation) were also used to analyse differences in agronomic traits, including dry weight, fresh weight, shoot length, shoot diameter, leaf length, leaf width, length/width ratio of leave, and leaf shape.

### 4.7. Acclimatization of Regenerated Plants

Nine-month-old plantlets (tenth generation) with well-developed shoots and roots were transplanted into 3-inch pots with peat moss and vermiculite (1:1) for acclimatization. The plants were incubated for acclimatization in a growth chamber with a 16/8 h (light/dark) photoperiod and a temperature of 25 ± 2 °C. A commercial powder fertilizer (Hyponex No. 5, N-P-K = 30-10-10, Hyponex Co., Marysville, OH, USA) was applied together with the irrigation at one-week intervals.

### 4.8. Determination of the Gastrodin Content

Leaves, roots and stems from 1.5-year-old plants of diploids and tetraploids (tenth generation) were harvested separately for analysis of the gastrodin content (mg/g DW). The tissues were dried and pulverised into powder, and extracted by distilled water (1 mL for 100 mg of fresh sample) for one day and then sonicated for 20 min. All extracts were centrifuged at 13,000 rpm for 10 min, and the supernatants were filtered by 0.22 μm PES syringe filters to remove the residues. The crude extracts were condensed to 5% of initial volume in a CentriVap Centrifugal Vacuum Concentrators (Labconco Corp., Kansas City, MO, USA). The fresh samples were collected and dried in the oven at 70 °C for calculating the ratio of dry and fresh weight. The system used for analysis of gastrodin was by under ultra-performance liquid chromatography (UPLC) system (ACQUITY UPLC, Waters Corp., Milford, CT, USA) with ACQUITY UPLC BEH C18 column (particle size 1.7 μm, 2.1 × 100 mm). The flow rate was at 400 uL/min, and a 10-min gradient was used for analysis. Two Mobile phases were used including solvent A contained 2% acetonitrile in water, and solvent B was acetonitrile. The extracts were separated by under the solvent gradient in the following manner: 0.5–99.5% of solvent B (0–4 min), 99.5% of solvent B (4–6 min), 99.5–0.5% of solvent B (6–6.5 min) and 0.5% of solvent B (6.5–10 min). The sample was reconstituted in H_2_O and injected 10μL into the UPLC system. The UPLC system was coupled online to the Waters Xevo TQ-S triple quadrupole mass spectrometer. The instrument was operated in negative multiple reaction-monitoring mode. The calibration curve was established using gastrodin (≥98% of purity) (SMB00313, Sigma-Aldrich Inc., St. Louis, MO, USA). Data acquisition and processing were performed using MassLynx version 4.1 and TargetLynx software (Waters Corp., Milford, CT, USA). The gastrodin compounds were eluted at 1.16 min (retention time) after injection, and their peak areas were calculated for quantification.

### 4.9. Determination of the Flavonoid Content

The total flavonoid content (mg/g DW) was evaluated using the aluminum chloride colorimetric method [38]. Leaves, roots, and stems from 1.5-year-old plants of diploids and tetraploids (tenth generation) were harvested separately for analysis. The tissues were dried and pulverised into powder and extracted by distilled water (1 mL for 100 mg of fresh sample) for one day and then sonicated for 20 min. All extracts were centrifuged at 13,000 rpm for 10 min, and the supernatants were filtered by 0.22 μm PES syringe filters to remove the residues. The crude extracts were used for determination of the flavonoid content. Each reaction contained 100 μL crude extract and 100 μL of 2% AlCl_3_·6H_2_O solution. After l hour of incubation at room temperature, the absorbance was determined at 415 nm by BioTek Eon microplate spectrophotometer (BioTek Instruments, Inc., Winooski, VT, USA). The calibration curve was established using rutin (0–200 mg/L) as the standard. Total flavonoid content is showed as mg rutin equivalents per g of dry weight.

### 4.10. Statistical Analysis

The experiment was designed with a randomized complete block design, and each treatment contained at least four replicates. The analysis of bioactive compounds was repeated for three times. Analysis of variance (ANOVA) was used for data evaluation. The significant differences among the treatments were compared using the Duncan multiple range test [39] with a 0.05 level of probability.

## Figures and Tables

**Table 1:** Effects of colchicine, *N*-(Phenylmethyl)-7*H*-purin-6-amine (*N*^6^-benzyladenine, BA), and 1-Phenyl-3-(1,2,3-thiadiazol-5-yl)-urea (thidiazuron, TDZ) on plantlet and subsequent polyploidy formation from the nodal-stem explants of *Anoectochilus formosanus*.

Colchicine (mg/L)	0.5 mg/L BA-Containing Medium		0.5 mg/L TDZ-Containing Medium	
	* Number of Survival Shoots	** Percentage of Polyploids (%)	Number of Survival Shoots	Percentage of Polyploids (%)
0 (control)	11.0 ± 1.2^a^ ***	0	14.5 ± 0.6^a^	0
100	5.3 ± 0.5^b^	10	8.3 ± 1.3^b^	50
250	2.5 ± 1.0^c^	20	3.0 ± 1.4^c^	20
500	1.5 ± 0.6^cd^	20	1.8 ± 0.5^cd^	20
1000	1.0 ± 0.0^d^	10	1.3 ± 0.5^d^	10

Four replicates (each contains five nodal-stem explants and each explant has three nodes) were used in each treatment. * Data were scored after three months of culture; ** Data were scored after one year of culture, 10 plantlets were selected randomly in each treatment to evaluate the polyploidy; *** Means ± Standard Deviation (SD) within a column followed by the same letter are not significantly different according to Duncan’s multiple range test (*P* ≤ 0.05).

**Table 2:** Ploidy level and morphological characteristics of vegetative clonal and seed-derived 4*x* plants of *Anoectochilus formosanus*.

Characteristics	Tenth Generation Vegetative Clonal 4*x* Plants Obtained from Nodal Stem Segments	Seed-Derived 4*x* Plants Obtained Via Self-Pollination
Ploidy level (cytometric patterns of young leaves, lateral shoots and root tips)	4*x* (all present 4C + 8C)	4*x* (all present 4C + 8C)
Stomata frequency (numbers/500 × 500 μm^2^)	28.8 ± 1.5^a^ *	28.5 ± 1.7^a^
Length of stomata (μm)	30.5 ± 1.3^a^	31.5 ± 1.3^a^
Width of stomata (μm)	26.5 ± 1.0^a^	26.3 ± 1.0^a^
Leaf shape	Cordate	Cordate

* Means ± SD within a row followed by the same letter are not significantly different according to Duncan’s multiple range test (*P* ≤ 0.05).

**Table 3:** Effect of ploidy level on growth and development of *Anoectochilus formosanus* in vitro.

Agronomic Traits	Ploidy Level	
	Diploid	Tetraploid
Dry weight (g)	0.41 ± 0.04^b^ *	0.76 ± 0.07^a^
Fresh weight (g)	0.97 ± 0.38^b^	1.73 ± 0.66^a^
Shoot length (cm)	2.85 ± 0.68^b^	3.76 ± 0.79^a^
Shoot diameter (cm)	0.30 ± 0.06^a^	0.31 ± 0.07^a^
Root length (cm)	1.22 ± 0.32^b^	2.96 ± 0.65^a^
Root diameter (cm)	0.16 ± 0.03^a^	0.21 ± 0.06^a^
Leaf length (cm)	1.87 ± 0.38^a^	1.76 ± 0.48^a^
Leaf width (cm)	1.35 ± 0.22^b^	1.88 ± 0.47^a^
Length/width ratio of leave	1.38 ± 0.21^a^	0.95 ± 0.13^b^
Leaf Shape	Ovate	Cordate

* Means ± SD within a row followed by the same letter are not significantly different according to Duncan’s multiple range test (*P* ≤ 0.05).

**Table 4:** Effect of ploidy level on characteristics of the stoma of *Anoectochilus formosanus*.

Characteristics	Ploidy Level	
	Diploid	Tetraploid
Stomatal density (no./500 × 500 μm^2^)	62.6 ± 3.6^a^ *	28.4 ± 3.3^b^
Stoma length (μm)	24.8 ± 1.4^b^	30.6 ± 0.6^a^
Stoma width (μm)	16.8 ± 1.4^b^	24.6 ± 0.6^a^
Length/width ratio of stoma	1.48 ± 0.09^a^	1.23 ± 0.01^b^
No. of chloroplasts per stoma	24.6 ± 2.0^b^	57.4 ± 1.7^a^

* Means ± SD within a row followed by the same letter are not significantly different according to Duncan’s multiple range test (*P* ≤ 0.05).

**Table 5:** The optimized parameters for analyzing gastrodin by the ultra performance liquid chromatography tandem mass spectrometry (UPLC-MS/MS) system.

Analyte	Precursor Ion (*m*/*z*)	Cone Voltage (V)	Product Ion (*m*/*z*)	Collision Energy (eV)
Gastrodin	285.1	10	105	20
			123 *	15
			161	5

* Represents the most abundant product ion which was applied for quantification.

## Abbreviations

BA	*N*-(Phenylmethyl)-7*H*-purin-6-amine, *N*^6^-benzyladenine
IBA	1 *H*-Indole-3-butanoic acid, Indole-3-butyric acid
MS	medium Murashige and Skoog (1962) medium
TDZ	1-Phenyl-3-(1,2,3-thiadiazol-5-yl)-urea, thidiazuron
